# Is Hyperopia an Important Risk Factor for PACG in the Dutch Population?—A Case Control Study

**DOI:** 10.1155/2013/630481

**Published:** 2013-09-15

**Authors:** Saskia H. M. van Romunde, Gijs Thepass, Hans G. Lemij

**Affiliations:** The Rotterdam Ophthalmic Institute, The Rotterdam Eye Hospital, P.O. Box 70030, 3000 LM Rotterdam, The Netherlands

## Abstract

*Objectives*. To determine if hyperopia is a risk factor for primary angle-closure glaucoma (PACG) in the Dutch population and to identify other biometrical parameters as risk factors for PACG including axial length (AL), anterior chamber depth (ACD), and *k* values. *Methods*. The study population consisted of PACG patients that had undergone a laser peripheral iridotomy (LPI). The control group consisted of age- and gender-matched cataract patients. The main outcome was hyperopia (spherical equivalent ≥+0.5 dioptres) measured with IOL Master or autorefractor. Refractive error, ACD, AL, and *k* values were tested with a Mann-Whitney *U* test and by logistic regression. *Results*. 117 PACG patients and 234 controls were included (mean age = 80 years ± 3.6). The prevalence of hyperopia in patients and controls was 69.6% and 61.1%, respectively (Fisher's test *P* = 0.076). Mann-Whitney *U* test showed no statistically significant relation with refractive error (*P* = 0.068) or *k* values (*P* = 0.607). In contrast, ACD and AL were statistically significant (*P* < 0.001). Tested with logistic regression, only ACD was a significant predictor of PACG (*P* < 0.001). *Conclusion*. There was no statistically significant correlation between refractive error and PACG. ACD was strongly correlated, though, with PACG, whereas AL turned out to be a less significant risk factor.

## 1. Introduction

Glaucoma is the most important cause of irreversible blindness in the world [1]. Primary angle-closure glaucoma (PACG) is highly prevalent in Asian countries. However, the Egna-Neumarkt Glaucoma Study stated that the burden of PACG in Europe has been underestimated previously [2]. The prevalence in this study was 0.6%, which accounts for about a quarter of all primary glaucoma cases. The most frequent type was chronic angle closure, which is more insidious and hence more often missed. Further damage of PACG can be easily prevented by performing a laser peripheral iridotomy (LPI) [3]. It is important to know more about the pathophysiology and risk factors for PACG to improve prevention. 

Several risk factors have been identified for PACG, including female gender, older age, and shallow anterior chamber depth (ACD) [2, 4–8]. Ophthalmologists in Europe commonly have the clinical impression that hyperopia is a risk factor for PACG. The relation between hyperopia and PACG was suggested as early as in 1970 [9]. Despite several studies, however, this relation has not been convincingly proven [4, 6–8]. A possible explanation for the mismatch between the clinical impression of such a relationship and the lack of evidence might be that most researches have been done in parts of Asia, where the anatomy of the eyes might be different from eyes in Europeans and myopia is more prevalent than in Europe [10]. Mechanisms for PACG vary among different ethnicities. Therefore, it has been suggested that mechanisms and risk factors for PACG should be studied in various populations [11]. To date, no studies on the risk factors for PACG have been done in Europe. Our study investigated the risk factors for PACG in the Dutch population. We set out with the assumption that hyperopia is an important risk factor for PACG. We wanted to determine if our assumption was right. In addition, we questioned whether other previously identified risk factors in other populations, including axial length (AL), ACD, and *k* values, play any role in the risk profile for PACG in the Dutch population. 

## 2. Methods

### 2.1. Study Population and Data Acquisition

The data was collected from all PACG patients that underwent an LPI in the Rotterdam Eye Hospital between 2008 and 2012 (*n* = 238). PACG was defined as an occludable angle as assessed upon gonioscopy together with structural or functional damage due to glaucoma in at least one eye. Evidence of glaucoma was investigated by fundoscopy, standard automated perimetry, and scanning laser polarimetry with the GDx (Carl Zeiss Meditec AG, Jena, Germany). Exclusion criteria were secondary glaucoma and narrow angles without physical or functional signs of glaucoma. Also, patients that underwent cataract surgery before an LPI were excluded because of the anatomical changes in the anterior segment caused by the surgery. The PACG patients that underwent cataract surgery after LPI treatment had been measured with the IOL Master V.4 (Carl Zeiss Meditec AG, Jena, Germany) before surgery. IOL Master measurements included refractive error, ACD, AL, and *k* values. This data was used for the analysis. The PACG patients that did not undergo any cataract surgery and had therefore not been measured with the IOL Master were asked to visit the hospital and undergo biometric assays for the current study. 

Control data was obtained from a database of all the cataract patients that underwent cataract surgery in the Rotterdam Eye Hospital in the period between 2007 and 2012 (*n* = 15118). This database contains the data of the IOL Master measurements performed before surgery. Incomplete data was removed from the dataset. To reach a power of 0.80, we selected two controls per PACG patient. In order to control for confounding factors, controls were matched by age and gender. Control subjects were listed anonymously by their randomly assigned patient ID number. Per PACG patient, we selected the first two control subjects on the list that matched in age and gender. One eye per patient was randomly selected by a randomization table. In some cases only one eye was suitable for the study. The same method was used for the control group. 

### 2.2. Additional Assessments

To minimize the burden on our patients, we did not recruit any patients that lived far away from the Rotterdam Eye Hospital. Other PACG patients that had not been previously measured with the IOL Master were invited for additional assessment. These were preferably combined with a regular visit. If no visit was planned during the course of the study, the patients were requested to visit the Rotterdam Eye Hospital for research purposes only. Informed consent was obtained before initiating the measurements. In these patients, the Lenstar LS900 (Haag-Streit AG, Köniz, Switzerland) was used for assessing ACD, AL, and *k* values, because the IOL Master was not available for these purposes. Three measurements were done in each eye. If a measurement failed due to eye movement or blinking, an additional measurement was made. The mean of the measurements was used for the analysis. Refractive error could not be measured with the Lenstar. Therefore, concurrently, an autorefractor ARK 530A (Nidek Co. Ltd. Gamagori, Japan) was used to assess any refractive error.

### 2.3. Statistical Analysis

All statistical analyses were performed with commercial software (SPSS version 21 for Windows, IBM, New York, USA). Statistical significance was assumed at *P* < 0.05 levels. Hyperopia was defined as a spherical equivalent ≥+0.5 dioptres (D) for the main analysis. Hyperopia is a common ocular condition. Hence, a spherical equivalent ≥+0.5 D may not be useful as a risk factor in clinical practice. Perhaps higher hyperopia might serve better to assess its role as a potential risk factor. Therefore, we redefined hyperopia in a subanalysis as a refractive error with a spherical equivalent ≥+3.0 D. Hyperopia was evaluated with the one-sided Fisher's test, odds ratios (OR), and its 95% confidence intervals (CI). Logistic regression was used for calculating a risk prediction for PACG based on refractive error. Refractive error, ACD, AL, and the mean of *k* values were evaluated for both the patients and the control group separately. The distributions of each parameter were tested for normality with the one-sample Kolmogorov-Smirnov test. Because not all data were parametric, any differences were tested conservatively with a Mann-Whitney *U* test. The results were further analysed by logistic regression.

## 3. Results

**Figure 1:**
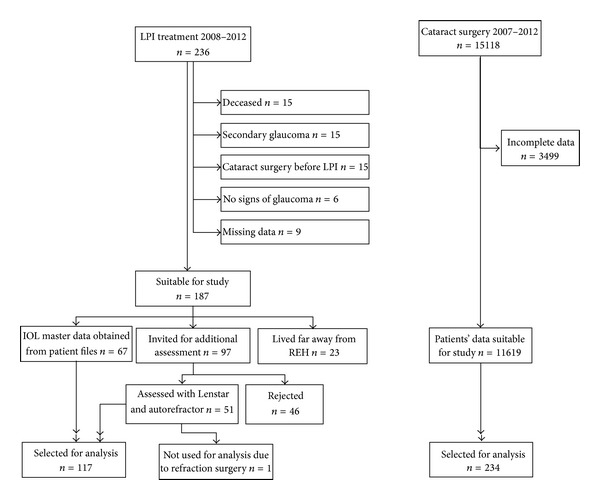
Flowchart for selecting PACG patients and controls. REH = Rotterdam Eye Hospital.

A total of 351 people were included in the study, 117 patients and 234 controls. The selection of the PACG patients has been presented in Figure 1.  Table 1 shows the demographics of the patients and control subjects. Fifty-eight percent of the participants were women. The mean age of the study population was 80 years. Sixty-seven PACG patients had been measured with the IOL Master, their data was obtained from patient files. Fifty patients were additionally assessed with the Lenstar and autorefractor. Out of the 117 patients, five patients had missing refractive error values. Twenty-five patients had only one eye suitable for the study because of cataract (*n* = 9), an LPI (*n* = 12), an enucleation (*n* = 2), trauma (*n* = 1), or a vitreous haemorrhage (*n* = 1) in one eye. The eyes of the other participants, in whom both eyes were eligible for inclusion, were randomly selected. 

### 3.1. Hyperopia

Table 2 shows the distribution of hyperopia in the PACG group and in the control group. It demonstrates that a high percentage of both patients and control subjects was hyperopic with an 8.5% difference between the two groups. This difference was not statistically significant (Fisher's test, *P* = 0.076). The odds ratios demonstrated no relation between PACG and hyperopia (OR 1.46; 95% CI 0.90–2.36). 

#### 3.1.1. Subanalysis

In the subanalysis, the cut-off value for hyperopia was chosen to be +3.0 D (spherical equivalent). The results have also been shown in Table 2. The Fisher test (*P* = 0.259) and OR demonstrated no statistically significant difference between the PACG group and the control group for the subanalysis. 

### 3.2. Risk Prediction

**Figure 2:**
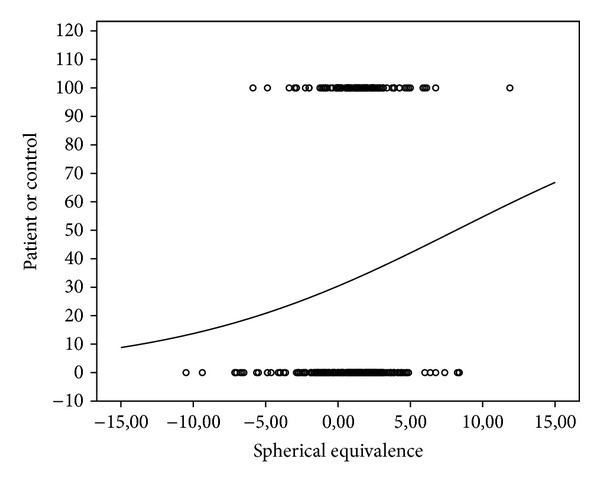
Distribution of spherical equivalent (in dioptres) among patients (*y* = 100) and controls eyes (*y* = 0). The curve represents the risk prediction of PACG based on spherical equivalent refractive error.

Figure 2 shows the distribution of the spherical equivalent in both the control group and the patient group. On the *y*-axis, the control subjects have been displayed at a value of 0 and the PACG patients at a value of 100. Most patients had a high refractive error. However, almost all of the patients' data overlapped those of the control subjects. The risk prediction has been integrated in Figure 2, where the maximum of 100 means a 100% chance of being a PACG patient. The curve is flat and the maximum does not reach the top. A clear cut-off value for a high chance of PACG could not be identified. This indicated that refractive error is not a strong predictor for PACG in this population. 

### 3.3. Other Biometrical Parameters

The means and standard deviations of all variables have been listed in Table 3. The Mann-Whitney *U* test was statistically significant for ACD (*P* < 0.001) and AL (*P* < 0.001). The results for refractive error, however, also for the *k* values, were not statistically significant (*P* = 0.068; *P* = 0.607). When we evaluated with logistic regression, only ACD remained statistically significant (*P* < 0.001).

## 4. Discussion 

To our knowledge, the current study is the first to present data about risk factors of PACG in a European population. We set out with the assumption that hyperopia would be a strong risk factor for PACG. However, the results demonstrated otherwise. Hyperopia was not statistically significantly more prevalent in the PACG group than in the control group. In the subanalysis, in which a higher hyperopia was investigated, the results also did not reach statistical significance. The refractive error turned out to be not a valuable predictor for PACG. Even if a person had a spherical equivalent of +10 dioptres—an extremely high refraction—the risk of PACG was only 54%. In our population, the overall prevalence of PACG was 33%, whereas in the normal population, the prevalence of PACG is approximately 0.6% [2]. Therefore, we can conclude that the risk prediction based on refractive error is of no value in a clinical setting.

A study dating back to 1970 stated that there is a relation between PACG and hyperopia [9]. In the 61 patients that the authors examined, both eyes were included in the study without statistically adjusting for any intereye correlation, thereby probably introducing a selection bias. They stated that the results were highly significant. However, there was an overlap from individual eyes between the PACG group and the group of control subjects. Moreover, the characteristics of the control group were not described. Therefore, the validity of the study may be questionable. 

Since then, several studies on PACG and refractive error have been performed in Asian countries. A relationship between PACG and hyperopia was found in a study done in an Indian urban area [12]. However, the same authors found contrary results in a research project that was performed two years later in a rural area in India [8]. Neither the Andhra Pradesh study nor the Namil study demonstrated a statistically significant relationship between PACG and hyperopia [6, 7]. It had been suggested by the authors that this was due to nuclear cataract, which would have been responsible for an increase in myopia and a decrease in hyperopia. This argument does not apply to our study, since our control group consisted of cataract patients. A difference between the methods of the mentioned studies and our study is that we matched the control subjects by age and gender, thereby controlling for potentially relevant confounding factors. 

The Beijing study found a relation between hyperopia and anterior chamber angle, suggesting that hyperopia is a predominant risk factor for PACG [13]. It has been suggested that the role of hyperopia is important for PACG because hyperopic eyes tend to have a larger lens/axial length factor (LAF) [14], and larger LAF is associated with PACG [15]. In our population, most of the PACG patients were hyperopic (Table 1). The reason why no significant difference between the two groups was found is that hyperopia was similarly prevalent in the control group as well. Though hyperopia may be involved in the pathogenesis of PACG, it cannot be accounted as a risk factor.

AL and ACD were the only variables that were significantly different in the two groups. ACD remained significant in the logistic regression, which means that out of the evaluated parameters, ACD is the most predicting factor for PACG. These results, obtained in a Caucasian population, correspond with the results of other studies [4–8, 12, 13].

### 4.1. Limitations

The current study has several limitations. The control subjects all had cataract, which may affect the refractive error, typically introducing a myopic shift, notably in nuclear cataract. Therefore, any myopic shift in our control group would have yielded a relative increase of the proportion of hyperopes in the PACG group. Nonetheless, no statistically significant relation between hyperopia and PACG was found. Hence, the results might be even less significant if our PACG group had been compared to healthy control subjects without cataract. Obviously, a prospective study in which healthy controls had been recruited would have been designed better. The advantage of the current study design, though, was that the biometric data of probably healthy eyes (except for their cataract) were readily available. A drawback of our methods was that the control subjects were not tested for PACG with gonioscopy. However, since the prevalence of PACG in the general population is estimated to be low, and probably similar to that in our control group, we assumed that this would not have significantly impacted on our results. 

The prevalence of hyperopia was higher in the PACG group than in the control group. The difference between the groups was not statistically significant. With a bigger sample size, smaller differences might have been detected. However, the clinical significance of detecting such a slight difference in refractive error is debatable.

The PACG patients had been treated with an LPI, which generally causes the angle to open. None of our outcome parameters, including the ACD, is affected by this treatment [16]. For our measurements, we used the IOL Master, the Lenstar, and the autorefractor. Several studies have investigated how well these instruments compare [17–20]. It turned out that the AL and *k* values are interchangeable, although the ACD is significantly different. Because the confidence intervals exceeded the difference, no adjustments had been made of the results. The primary objective of this study was refractive. If the focus of a future study is on ACD, only one type of measurement device should be used better.

## 5. Conclusion

Although it is commonly believed that hyperopia is a risk factor for PACG, we found no such evidence in the current study. ACD was the only statistically significant risk factor that could be identified. AL turned out to be a statistically weaker risk factor than ACD. Since PACG is an underestimated disease in Europe and preventive measures are relatively easy to perform, clinicians should be aware that nonhyperopic eyes also run the risk of angle closure. This risk calls for gonioscopy in all eyes, regardless of any refractive error. In addition, more research into hyperopia is needed. 

## Figures and Tables

**Table 1:** Demographics of the study population and instrument used for measurements.

	Patient (*n* = 117)	Controls (*n* = 234)
Mean age	80 (76.4–80.6 95%-CI)	80 (76.4–80.6 95%-CI)
Gender		
Women	65 (58%)	130 (58%)
Men	48 (42%)	96 (42%)
Instrument		
IOL Master	67 (59%)	234 (100%)
Lenstar + AR	46 (41%)	—

AR: autorefractor.

**Table 2:** Hyperopia defined as a refractive error spherical equivalent (SE) ≥ 0.5 dioptres (D) for the main outcome and SE ≥ 3.0 D for a subanalysis. Odds ratios (OR) and their 95% confidence intervals (CI) are shown. Results of the one-sided Fisher's test *P* < 0.05 were assumed to be significant.

	Patient (*n* = 112)	Control (*n* = 234)	OR (95% CI)	Fisher's test
Hyperopia ≥ 0.5 D				
Absent	34 (30.4%)	91 (38.9%)	1	*P* = 0.076
Present	78 (69.6%)	143 (61.1%)	1.460 (0.90–2.36)	
Hyperopia ≥ 3.0 D				
Absent	92 (82.1%)	200 (85.5%)	1	*P* = 0.259
Present	20 (17.9%)	34 (15.6%)	1.279 (0.70–2.34)	

**Table 3:** Mean and standard deviation (SD) of the variables refractive error spherical equivalent (SE) in dioptres (D), anterior chamber depth (ACD) in millimetres (mm), axial length (AL) in mm, and mean of *k* values in diopters. Differences were tested with the Mann-Whitney *U* test (MWU). Results *P* < 0.05 were assumed to be significant.

	Patient (*n* = 117)		Controls (*n* = 234)		MWU	Logistic regression
	Mean	SD	Mean	SD		
SE	1.38	2.37	0.67	2.82	*P* = 0.068	*P* = 0.766
ACD	2.71	0.28	3.08	0.38	*P* < 0.001	*P* < 0.001
AL	22.86	1.03	23.47	0.08	*P* < 0.001	*P* < 0.671
*k* values	43.98	3.26	43.86	0.87	*P* = 0.607	*P* = 0.819
